# The N-terminal autoinhibitory module of the A1 domain in von Willebrand factor stabilizes the mechanosensor catch bond†

**DOI:** 10.1039/d2cb00010e

**Published:** 2022-04-07

**Authors:** Yunduo Charles Zhao, Haoqing Wang, Yao Wang, Jizhong Lou, Lining Arnold Ju

**Affiliations:** School of Biomedical Engineering, Faculty of Engineering, The University of Sydney Darlington NSW 2008 Australia arnold.ju@sydney.edu.au; Charles Perkins Centre, The University of Sydney Camperdown NSW 2006 Australia; Heart Research Institute, Newtown NSW 2042 Australia; Cellular and Genetic Medicine Unit, School of Medical Sciences, University of New South Wales NSW 2052 Australia; Key Laboratory of RNA Biology, CAS Center for Excellence in Biomacromolecules, Institute of Biophysics, Chinese Academy of Sciences Beijing 100101 China; The University of Sydney Nano Institute (Sydney Nano), The University of Sydney, Camperdown NSW 2006 Australia; Coulter Department of Biomedical Engineering, Georgia Institute of Technology, Atlanta GA 30332 USA

## Abstract

The von Willebrand factor (VWF), by interacting with the circulatory system and platelets, harnesses hemodynamic forces to form hemostatic plugs or occlusive thrombi. The autoinhibitory modules (AIMs) flanking the VWF-A1 domain were found to contribute to its biomechanical activation. However, how AIM sequences regulate the VWF-A1 binding behavior is controversial and incompletely understood as their structures are currently unsolvable by crystallography. To address this, we first performed molecular dynamics simulations to predict the N-terminal AIM (N-AIM; residues Q1238–E1260) structure. Excitingly, we found that N-AIM could cooperate with C-AIM to form a joint Rotini-like structure, thereby partially autoinhibiting the VWF-A1–GPIbα interaction. Furthermore, we used biomembrane force probe (BFP) assays to experimentally demonstrate that the VWF-A1 containing long N-AIM sequence (1238-A1) exhibited catch-bond behavior as the force first decelerated (catch) and then accelerated (slip) the dissociation. Conversely, VWF-A1 with short N-AIM (1261-A1) displayed bi-variable behaviors with either catch (1261^H^-A1) or slip bonds (1261^L^-A1). Notably, such bi-variable transition happened at low temperatures or high pH levels, whereas Q1238–E1260 stabilized the 1238-A1 catch bond regardless of the environmental factors. The physiological study was complemented by platelet perfusion assays using microfluidics. Taken together, these studies provide new mechanobiology on how N-AIM serves as a mechano-regulator of VWF activity, which inspires future VWF-A1 dependent antithrombotic approaches.

## Abstract 2

The N-AIM of VWF-A1 forms a Rotini-like structure, therefore partially autoinhibit VWF-A1–GPIbα interaction. The N-AIM acts as a defending sword to protect and stabilize the VWF-A1 structure under harsh environments.

## Graphical abstract

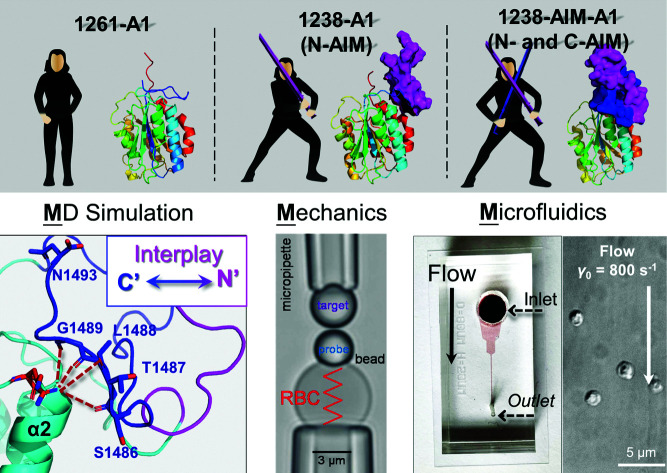

## 1. Introduction

**Fig. 1:**
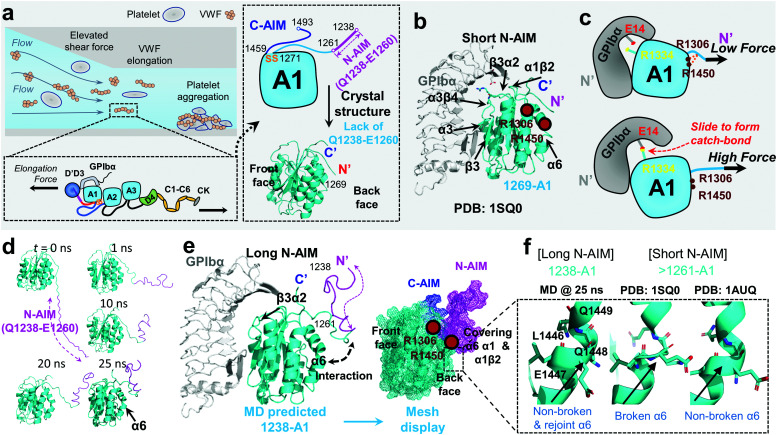
VWF mechanosensing biophysics and structural insights into the A1 N-terminal autoinhibitory module (N-AIM). (a) Top left: VWF biomechanical activation. Upon vessel injury or stenosis, elevated shear rate elongates VWF and promotes biomechanical platelet aggregation; bottom left: schematic of VWF domain organization. Elongational force relieves VWF-A1 autoinhibition thereafter exposes its binding site to the platelet GPIbα; right: the zoom-in structure of the VWF-A1 domain and its flanking AIMs. The disulfide bond of the VWF-A1 domain (orange), flanking N-AIM consisting of residues D1261–Y1271 (cyan) and Q1238–E1260 (magenta), and C-AIM (blue) are indicated. The 1269-A1 structure (PDB 1SQ0^16^) is used. (b) The co-crystal structure of short N-AIM A1–GPIbα (PDB 1SQ0^16^). Note that VWF-A1 has two GPIbα binding interfaces at the front face: the larger interface resides in α3 helix, β3 strand, and α3β4 loop; the smaller interface resides in α1β2, β3α2, and α3β4 loops. Residues R1306 and R1450 (dark red) were reported to be covered by N-AIM using HDX experiments.^41^ (c) The sliding–rebinding mechanism explaining the VWF-A1–GPIbα catch bond. The key residues for catch bond behavior are VWF-A1 R1334 (yellow) and GPIbα E14 (red). Note that the breakage of the A1 D1269–R1306/R1450 salt bridges enables the A1 R1334 slide then rebinds GPIbα E14 as a stronger interaction upon force pulling.^12^ (d) Sequential snapshots of representative free MD-simulated structures showing the 1238-A1 (cyan) interplay with N-AIM (Q1238–E1260; magenta). At *t* = 25 ns, N-AIM forms a Rotini-like structure that interacts with VWF-A1 α1/α6 helices. (e) Left: the complexed structure of our MD predicted 1238-A1 with GPIbα; right: a mesh display of our MD predicted 1238-A1, which shows the N-terminal (magenta mesh) and C-terminal (blue mesh) AIM cooperatively covering the back face of the VWF-A1 domain (cyan mesh). (f) The non-broken and rejoint (MD predicted 1238-A1), broken (PDB 1SQ0)^16^ and non-broken (PDB 1AUQ)^42^ α6 helices in long and short N-AIM A1 structures.

von Willebrand factor (VWF) is a multimeric plasma protein that mediates platelet adhesion as a key event in hemostasis and thrombosis. Intriguingly, force and VWF function are so closely intertwined, enabling its rapid activation in response to elevated hemodynamic forces in arterial blood flow (Fig. 1a, top left).^1^ In structure, each VWF monomer of 250 kDa consists of D1, D2, D′D3 assembly, A1A2A3 domain, D4 assembly, C domains, and CTCK domain in order from the N-terminus according to the latest definition^2^ (Fig. 1a, bottom left) and forms homodimers as Pro-VWF *via* disulfide bridges between cysteine residues located in the C-terminus.^3^ The mechanosensing and force-induced transition of VWF were recognized at its A1,^4^ A2,^5,6^ D4,^7^ and C domains,^8^ where the A1 domain displays a unique force-enhanced binding kinetics interacting with the platelet receptor glycoprotein Ibα (GPIbα).^4,9–15^ Revealed by the crystalized structure, the VWF-A1 domain binds GPIbα with two contact sites at the front face of the VWF-A1–GPIbα interface (Fig. 1b): one proximal to the A1 N/C-termini that involve α1β2, β3α2 and α3β4 loops and the other distal one that spans α3 helix, β3 strand, and α3β4 loop.^16,17^ Clinically, targeting the VWF-A1–GPIbα axis represents a new antithrombotic therapeutic strategy.^1,18^ Anti-VWF caplacizumab (ALX-0081) has been recently approved by the FDA in 2019 to treat thrombotic thrombocytopenic purpura (TTP).^19^ Anti-VWF-A1 aptamer ARC1779^20^ and anti-GPIbα anfibatide^21^ have recently entered clinical trials on patients with TTP and acute coronary syndrome.^1,18^ Nevertheless, targeting VWF–GPIbα still raises concerns on severe bleeding side effects as most existing antithrombotics do.^1,18^ To this end, the investigation into the biomechanical regulation of VWF function promises new insights to optimize VWF–GPIbα targeted therapeutics.

With a unique mechanosensitive feature, VWF–GPIbα mediated platelet adhesion is enhanced by flow and shear rates.^22^ The current view indicates that this counterintuitive phenomenon is related to the autoinhibitory mechanism of VWF activation. In normal physiological circulation, the plasma VWF has a limited binding capacity with platelet GPIbα as the A1 domain is covered by its flanking autoinhibitory modules (AIMs),^13^ as well as the adjacent D′D3 assembly^23^ and the A2 domain.^24–26^ Specifically, the N-terminal AIM or N-AIM sequence refers to the residues Q1238–Y1271,^4,13,27^ and the C-terminal AIM or C-AIM consists of the residues D1459–N1493 (Fig. 1a, right).^13,28^ Once VWF is immobilized onto subendothelial collagen and subjected to an elevated shear rate due to vessel injury and stenosis,^29,30^ hemodynamic drag forces on VWF elongate the macromolecule to adopt an extended conformation (Fig. 1a, top left).^31,32^ Therefore, VWF-A1 autoinhibition is relieved and thereafter its binding site to platelet GPIbα (mechano-presentation) is exposed.^6,33^

Recent advances in dynamic force spectroscopy (DFS) techniques, such as atomic force microscopy (AFM),^12^ biomembrane force probe (BFP),^4,10,34^ and optical tweezers (OT),^11,13,35^ enabled the characterization of force-dependent VWF–GPIbα binding kinetics at the molecular scale. To complement such biophysical studies, shear-dependent platelet perfusion assays were performed using microfluidics.^4,10,12,15^ Respective DFS and perfusion flow studies on various VWF-A1 constructs containing AIM sequences of different lengths are summarized in Table 1. It is reported that the A1 domain is capable of representing the full-length VWF–GPIbα interaction at the force regime from 10–70 pN and a wall shear rate of 800 s^−1^ (Table 1 and Fig. S1, ESI†).^4,12^ The most counterintuitive finding on mechano-regulation of VWF-A1 binding is that the force strengthens the VWF-A1–GPIbα interaction to exhibit ‘catch-bond’ behavior (force prolongs the bond lifetime and decreases the off-rate).^4,9–15^ Notably, increasing evidence suggests that A1 AIM flanking sequences play key roles in catch-bond behavior and the related flow-enhanced VWF adhesive functions.^11,13,27,36,37^

**Table 1:** A summary of the biophysical studies on VWF-A1–GPIb interactions. Respective listed columns are: details of starting and ending residues, production source of recombinant VWF-A1, corresponding PDB structure codes if applicable (resolved sequence of VWF-A1 indicated), assay types, physical parameter ranges, as well as VWF-A1 bond behaviors and functional phenotypes. Most of these studies exhibited force-strengthened, flow-enhanced, and catch-bond like platelets-GPIbα binding phenotypes. The perfusion flow assays (PFA) examined the VWF-A1–GPIbα mediated platelet adhesion with whole blood (WB), washed platelets (PLT), or reconstituted blood (RB) perfusion.^29,57^ Studies evaluated by shear stress *τ* are converted to shear rate *γ* by the relationship *τ* = *γ* × *μ*, where the dynamic viscosity *μ* of PLT is considered to be 100 dyn s cm^−2^. The surface plasmon resonance spectroscopy (SPR) and HDX mass spectrometry characterized the VWF-A1–GPIbα interaction at zero force.^41,46,55^ DFS measurements with AFM, BFP, and OT characterized VWF-A1–GPIbα binding kinetics at a range of applied forces^4,11,12^

N-to-C residue numbering of the VWF-A1 constructs	Production source	PDB code with resolved A1 residues	Assay types	Physical parameter ranges	Phenotype	Ref.
Long N-AIM (starts ≤Q1238)						
Q1238–P1471	*Escherichia coli*	N/A	PFA	100–10 000 s^−1^ (PLT)	Flow-enhanced adhesion, catch-bond like	43
				300–10 000 s^−1^ (WB)	Flow-enhanced adhesion, catch-bond like; autoinhibition by N-AIM	15
				1–10 000 s^−1^ (PLT); 1500 s^−1^ (WB)	Flow-enhanced adhesion, catch-bond like; autoinhibition by N-AIM	4
				80–1600 s^−1^ (PLT)	Flow-enhanced adhesion, catch-bond like	10
				1500 s^−1^ (WB)	Autoinhibition by A2	24
			BFP	0–80 pN	Catch bond	44 and 45
					Catch bond; autoinhibition by N-AIM	4
		1AUQ (resolved D1261–T1468)	SPR	80–1600 s^−1^ (PLT)	Flow-enhanced adhesion, catch-bond like; autoinhibition by N-AIM and C-AIM	46
			PFA	800 s^−1^ (WB)		
			BFP	5–50 pN	Catch bond; autoinhibition by A2	47
		1M10 (resolved H1268–P1466)	PFA	20–1600 s^−1^ (PLT)	Flow-enhanced adhesion, catch-bond like	48
Q1238–D1472		N/A	PFA	20–16 000 s^−1^ (PLT)	Flow-enhanced adhesion, catch-bond like	49
				25–400 s^−1^ (PLT)		50
S1208–V1496		N/A	PFA	50–1500 s^−1^ (WB)	Flow-enhanced adhesion, catch-bond like; autoinhibition by D′D3	51
G1242–P1480		1SQ0 (resolved D1269–P1466)	AFM	0–120 pN	Catch bond; autoinhibition by N-AIM	12
			PFA	10–10^7^ s^−1^ (PLT)	Flow-enhanced adhesion, catch-bond like; autoinhibition by N-AIM	
		N/A	PFA	300–1500 s^−1^ (PLT)	Flow-enhanced adhesion, catch-bond like	40
Q1238–G1874 (A1A2A3)	Mammalian cells (HEK293)	1AUQ (resolved D1261–T1468)	PFA	1500 s^−1^ (WB)	Flow-enhanced adhesion, catch-bond like; autoinhibition by N-AIM	27
T1248–T1487		N/A	PFA	0–9600 s^−1^ (PLT)	Flow-enhanced adhesion, catch-bond like; autoinhibition by N-AIM	52
Q1238–D1472		N/A	OT	0–200 pN	Benchmarked rupture force of VWF-A1 *vs.* full-length VWF	53
Full-length VWF		1AUQ (resolved D1261–T1468)	PFA	500–4000 s^−1^ (PLT)	Flow-enhanced adhesion, catch-bond like; autoinhibition by A2	26
S1208–V1496	*D. melanogaster*	N/A	PFA	3000–25 000 s^−1^ (WB & PLT)	Flow-enhanced adhesion, catch-bond like	54
Q1238–D1472	Baby hamster kidney-derived cells (BHK)	1SQ0 (resolved D1269–P1466)	HDX	N/A	N-AIM partially covered VWF-A1 at α1/α6 helices and α1β2/β3α2 loops; autoinhibition by N-AIM and C-AIM	13, 28 and 41
Q1238–N1493		1AUQ (resolved D1261–T1468); 7A6O (resolved I1262–P1466)	OT	0–70 pN	AIM (N-AIM & C-AIM) unfolded by force, autoinhibition relieved; joint autoinhibition by N-AIM and C-AIM	13

Short N-AIM (starts ≥D1261)						
D1261–P1471	*Escherichia coli*	1AUQ (resolved D1261–T1468)	PFA	300–10 000 s^−1^ (WB)	Flow-enhanced adhesion, catch-bond like; autoinhibition by N-AIM	15
		N/A	BFP	0–80 pN	Slip bond	4
			PFA	1–10 000 s^−1^ (PLT); 1500 s^−1^ (WB)	Flow-abolished adhesion, slip-bond like	
E1260–T1468		N/A	SPR	N/A	Flow-enhanced adhesion, catch-bond like	55
E1260–G1479		N/A	PFA	200–1500 s^−1^ (PLT)	Flow-enhanced adhesion, catch-bond like	56
D1261–G1874 (A1A2A3)	Mammalian cells (HEK293)	1AUQ (resolved D1261–T1468)	PFA	1500 s^−1^ (WB)	Flow-enhanced adhesion, catch-bond like; autoinhibition by N-AIM	27
D1261–D1472		1U0N (resolved D1261–T1468)	PFA	10–3000 s^−1^ (PLT)	Flow-enhanced adhesion, catch-bond like; autoinhibition by N-AIM	36
D1261–P1466		N/A	OT	0–60 pN	Slip bond	38
I1262–P1466		N/A	OT	0–40 pN	Double slip bond	11
D1261–D1472	Baby hamster kidney-derived cells (BHK)	1SQ0 (resolved D1269–P1466)	HDX	N/A	Non-significant coverage observed	13,28,41
H1268–N1493		1AUQ (resolved D1261–T1468); 7A6O (resolved I1262–P1466)	OT	0–70 pN	AIM (N-AIM & C-AIM) unfolded by force, autoinhibition relieved; autoinhibition by C-AIM	13

As summarized in Table 1, various recombinant VWF-A1 constructs used in past studies were derived and categorized into two classes: one with a short N-AIM sequence, *e.g.*, 1261-A1 (D1261–D1472),^11^ and the other with a long N-AIM sequence, *e.g.*, 1238-A1 (Q1238–D1472).^12^ DFS experiments have found that the VWF-A1–GPIbα catch bond is associated with the 1238-A1 construct, while converted into a slip bond (force shortens the bond lifetime and increases the off-rate) with the 1261-A1 construct.^4,10,38^ Controversially, other studies demonstrated that 1261-A1 exhibits a catch bond and a stronger interaction with GPIbα than 1238-A1 at high forces.^13,15,27,36^ For narrative convenience, we term the low (slip bond) and high (catch bond) binder forms as 1261^L^-A1 and 1261^H^-A1, respectively. Although 1261^L^-A1 and 1261^H^-A1 have the same amino acid sequences (D1261–D1472),^4,15,39,40^ the bi-variable behaviors when interacting with GPIbα (Table 1) suggest that they may have different phenotypes.

To explain the structural basis of VWF-A1–GPIbα on catch–slip bonding, steered molecular dynamics (SMD) simulations were performed on the 1269-A1 structure (PDB 1SQ0), with a short N-AIM A1 that starts at residue D1269. This computational study proposed a sliding–rebinding mechanism in which the increasing force breaks the A1 D1269–R1306/R1450 salt bridges between the N-AIM and α1/α6 helices and generates a torque to rotate the A1 domain (Fig. 1c).^12^ The subsequent relative sliding enables the new formation of a strong long-lived salt bridge between A1 R1334 (on β3α2 loop) and GPIbα E14 resides at the N-AIM proximal binding interface (Fig. 1c). Notably, the interaction of the N-AIM–α1/α6 helices at the A1 back face is critical to catch bond behavior, given that two type 2B von Willebrand disease (VWD) mutations, R1306Q and R1450E, were shown to cause A1 R1334–GPIbα E14 bond formation at zero force, thereby exhibiting an ordinary slip bond.^12^ A recent study using hydrogen–deuterium exchange (HDX) mass spectrometry has shown that the 1238-A1 sequence Q1238–E1260 covers the residues R1306 on the α1 helix and R1450 on the α6 helix (Fig. 1b and e).^41^ In the presence of C-AIM, the AIMs cover more residues, including the α2 helix and even the β3α2 loop, one of the VWF-A1–GPIbα binding sites. This experimental evidence raised a hypothesis that the N-AIM sequence Q1238–E1260 regulates the VWF-A1–GPIbα interaction under low force conditions and may further stabilize the sliding–rebinding potential for catch bonds, while 1261-A1 has unstable N-AIM–α1/α6 interplay, leading to variable catch–slip bonding behaviors.^13^ Nevertheless, there is no definitive structural evidence that explains how the long N-AIM sequence regulates the VWF-A1–GPIbα catch bond behavior and the related autoinhibition of VWF function.

To this end, the present study combined MD simulation, BFP, and microfluidic perfusion assays as a multidisciplinary approach, which reveals the structural and functional basis of the N-AIM of the A1 domain in stabilizing the VWF-A1–GPIbα interaction and regulating the VWF binding mechanosensitivity.

## 2. Results

### 2.1 Molecular dynamics simulation predicts the 1238-A1 structure

Although the previous DFS experiments demonstrated the essential role of N-AIM in maintaining and regulating the VWF-A1–GPIbα catch bond (Table 1 and Fig. 1c), detailed structural insights into N-AIM mechano-regulation are elusive. It is largely because most of the existing VWF-A1 structures only include short N-AIM sequences (*e.g.*, 1261-A1 and 1269-A1; Table 1). In certain studies, although long N-AIM A1 constructs were used, residues Q1238–E1260 did not assume a stable structure, therefore did not appear in the final resolved structures (Fig. 1a and b).^13^ To this end, using MD simulations, for the first time we computationally predicted the 1238-A1 structure with the N-AIM sequence Q1238–E1260 (Fig. 1d). At 1–10 ns, Q1238–E1260 started folding, formed more bonds, then rotated counterclockwise by 45° (Fig. 1d, magenta). From 20 to 30 ns, the 1238-A1 was stabilized without major changes in all three 30 ns independent MD simulations. The N-AIM Q1238–H1265 formed a Rotini-like structure (Fig. 1d and e), which partially covered the back face of 1238-A1, spanning residues R1306–R1308 and Q1448–D1459 on the α1/α6 helices, respectively. This coverage involves residues R1306 and R1450, which are essential for the VWF-A1 catch bond mechanism.^12^ Notably, similar N-AIM coverage onto VWF-A1 was suggested by Auton *et al.* using anti-VWF-A1 antibody A108 with an overlapped epitope^27^ and was then independently confirmed by Deng *et al.* using HDX mass spectrometry.^28^ This consistency validates our MD predicted 1238-A1 structure for further analysis.

To provide structural insights on how the lack of N-AIM Q1238–E1260 sequences leads to the distinct binding phenotypes for 1261-A1, we compared two existing short N-AIM A1 structures, *i.e.*, PDB 1SQ0*vs.*1AUQ (Fig. 1f, middle and right). Two distinct α6 helix phenotypes were found and termed as broken (1SQ0) or non-broken (1AUQ) according to the presence of ruptured α6 helical hydrogen bonds. Notably, the broken α6 helix from the original crystal structure (1SQ0) was rejoined (convert from broken to non-broken) after being linked to N-AIM in our MD simulation on 1238-A1 (Fig. 1f, left).

### 2.2 Establishing a biomembrane force probe to characterize VWF-A1–GPIbα binding

**Fig. 2:**
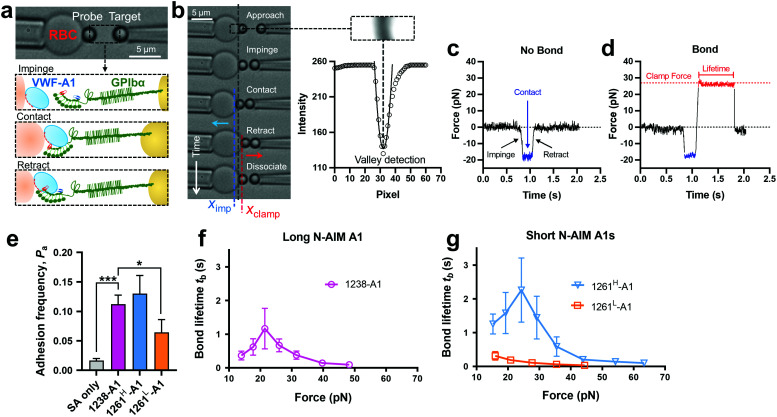
BFP measurement of binding between GPIbα and various VWF-A1s. (a) BFP photomicrograph. A micropipette-aspirated RBC with a bead (left, termed “probe”) glued to the apex, thereby formed a pico-force sensor (spring constant is set to 0.3 pN nm^−1^ by default). The probe bead was then aligned with another bead (right, termed “target”) aspirated using an apposing micropipette. VWF-A1 and GPIbα were covalently linked to the probe (left) and target (right) beads, respectively. During the BFP test cycle, GPIbα on the target bead impinged VWF-A1 to let binding sites contact, then retracted and subsequently formed bonds visualized in force spectroscopy. (b) The deflection of the RBC, and positions of probe and target beads in a test cycle of force clamp assay. The tracking zone for the RBC–probe is indicated. The edges of the RBC and probe bead were tracked by a valley detection algorithm. In each cycle, the GPIbα coated target bead was driven by a piezo actuator and approached the probe bead (∼0 pN), contacted for a certain time duration with a 20 pN impingement force (blue dashed line), retracted and ended the cycle if ‘no bond’ (c) or held at a preset force (red dash line) until dissociation (signified by a force drop to zero) if ‘bond’ was detected (d). (c) and (d) Force *vs.* time traces from ‘no bond’ and ‘bond’ events. Bond lifetime was measured across the interval between the instant force reached the clamp force level and the instant of bond dissociation. (e) Binding specificity. Adhesion frequencies (*P*_a_) between the GPIbα targets and probes coated without (SA only) or with indicated ligands (1238-A1, 1261^H^-A1, and 1261^L^-A1).^4^ Each probe–target pair was tested repeatedly for 200 approach–contact–retract cycles to estimate *P*_a_. Five probe–target pairs were tested to obtain mean ± S.E.M. * = *p* < 0.05; *** = *p* < 0.001, assessed by unpaired, two-tailed Student's *t*-test. (f) and (g) binding phenotypes of various VWF-A1 constructs interacting with GPIbα. Plots of lifetime *vs.* force were compared between the “Long N-AIM A1” (f) and “Short N-AIM A1” (g). The lifetimes (mean ± S.E.M. of >20 measurements per point) of 1238-A1–GPIbα (magenta), 1261^H^-A1–GPIbα (cyan) and 1261^L^-A1–GPIbα (orange) were measured by the force-clamp assay at each force.

Towards a further understanding of the structure–function relationship with respect to the N-AIM of VWF-A1, we employed the DFS technique—BFP to characterize the VWF-A1–GPIbα interaction in a bead–bead mode as previously established.^58^ VWF-A1 was coated on a glass bead (probe) attached to the apex of a micropipette-aspirated red blood cell (RBC) (Fig. 2a and b, left), and GPIbα was immobilized on another glass bead (Target) aspirated using an apposing micropipette (Fig. 2a and b, right). In experiments, the target bead repeatedly underwent approach, impinge, contact, retract and dissociate stages towards the probe bead in each BFP touch cycle (200 cycles in total; Fig. 2b). By tracking the RBC–probe edge positions using the valley detection algorithm (Fig. 2b), BFP calculated the holding force and generated force spectroscopy traces (force *vs.* time in Fig. 2c and d). After a controlled contact, the BFP detected ‘no bond’ (Fig. 2c) or ‘bond’ (Fig. 2d) from the force signal deflection upon target retraction and calculated adhesion frequency (*P*_a_) from repeated touches (Fig. 2e). Binding specificity was established for VWF-A1. Specifically, the GPIbα-bearing target bead adhered at significantly higher frequencies to the probe bead coated with VWF-A1s (1238-A1, 0.112 ± 0.003; 1261^H^-A1, 0.130 ± 0.031; 1261^L^-A1, 0.065 ± 0.022) than nonspecific controls (Streptavidin or SA only, 0.017 ± 0.003).

### 2.3 BFP and microfluidic perfusion assays benchmark binding phenotypes of various VWF-A1 constructs

Of note, Tischer *et al.* reported that platelets translocated slower on 1261-A1 than 1238-A1 in a range of wall shear rates,^15^ which contradicts our observation that 1238-A1 (from Cruz *et al.*^39^) supported more stable translocation than 1261-A1.^4^ These discrepant results suggested that different binding states may exist for 1261-A1 constructs (*i.e.*, low binder 1261^L^-A1 and high binder 1261^H^-A1). Hereby, we collected both 1261-A1 constructs (1261^H^-A1 from Tischer *et al.*^15^ and 1261^L^-A1 from Cruz *et al.*^4^) and used BFP to benchmark their binding phenotypes. We observed that 1261^H^-A1 dissociated from GPIbα as a catch bond (Fig. 2g, cyan), exhibiting a similar qualitative pattern but a quantitatively longer bond lifetime (peak bond lifetime at 20–25 pN, 2.26 ± 0.95 s) than that of 1238-A1 (peak bond lifetime at 20–25 pN, 1.16 ± 0.60 s; Fig. 2f, magenta). In sharp contrast, 1261^L^-A1 dissociated from GPIbα as a slip bond (Fig. 2g, orange), consistent with the result in our previous study.^4^ Together, the BFP data suggested that 1261^H^-A1 and 1238-A1 share a similar catch-bond mechanosensory mechanism, but autoinhibition was partially relieved with short N-AIM A1.^15^ In contrast, 1261^L^-A1 formed a monophasic slip-only bond with GPIbα, demonstrating the possible structural variation that abolishes the catch-bond behavior.

**Fig. 3:**
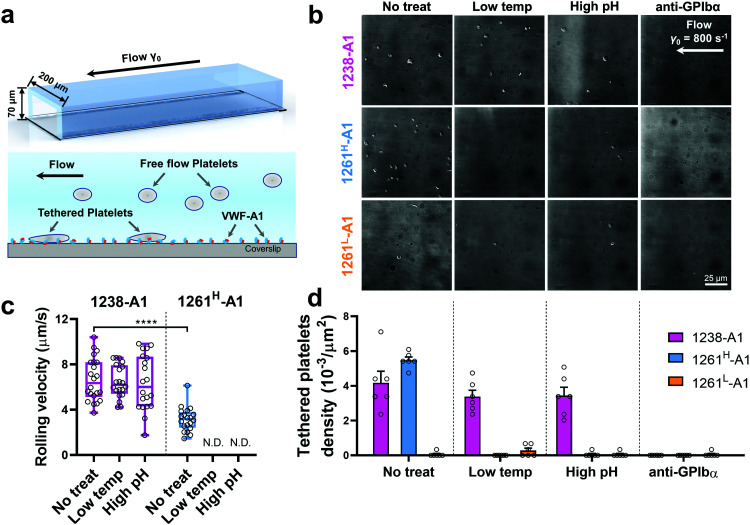
Functionality variations in supporting platelet adhesion by different VWF-A1 preparations. (a) Schematic of microfluidic perfusion assays. The washed platelets were perfused over the microfluidic channel at a wall shear rate of *γ* = 800 s^−1^. The bottom coverslip was precoated with VWF-A1 ligands at a concentration of 50 μg mL^−1^. Platelets would tether to the surface of a coated coverslip under high shear conditions if a catch bond was formed between GPIbα and VWF-A1s. (b) Representative snapshots of tethered platelets (bright white objects) in a microfluidic channel. Prior to incubating with microfluidic channel surfaces, 1238-A1 (1st row), 1261^H^-A1 (2nd row), and 1261^L^-A1 (3rd row) were subjected to: no treatment (1st column); low temperature, frozen under −80 °C overnight, then thawed (2nd column); incubated at a high pH level (9.6) with an adjusted Tyrode buffer for 24 h (3rd column); and addition of an anti-GPIbα antibody (clone ALMA12; 10 μg mL^−1^)^64^ to block GPIbα mediated platelet tethering (4th column). The photomicrographs depict the platelets adhered to the surface during perfusion and each represents experiments with two different platelet donors. (c) Rolling velocities of the platelet in the region of interest (ROI) during the 30 s perfusion at a wall shear rate of *γ* = 800 s^−1^ for VWF-A1s under no treatment and low temperature or high pH level treatment. Data are presented as box plots of a total of six ROIs selected from two independent experiments. **** = *p* < 0.0001, assessed by unpaired, two-tailed Student's *t*-test. N.D. = not detectable. (d) Tethered platelet density, in the ROI during 30 s perfusion at a wall shear rate of *γ* = 800 s^−1^ for VWF-A1s under no treatment, low temperature, high pH level, or anti-GPIbα blocking conditions. Data are presented as mean ± S.E.M. of a total of six ROIs selected from two independent experiments.

We then performed the microfluidic perfusion assay that is widely used to recapitulate platelet adhesion under blood flow.^4,12,15,59,60^ The washed platelets were perfused through a microfluidic channel with a width of 200 μm and a height of 70 μm at an arterial wall shear rate of *γ* = 800 s^−1^ (Fig. 3a, top).^29^ The bottom coverslip of the microfluidic channel was coated with 1238-A1, 1261^L^-A1, or 1261^H^-A1. Tethered platelet density, defined as the number of tethering and rolling platelets in the field of view over 30 s perfusion,^57,61^ was determined to indicate the cellular on-rates of VWF-A1–GPIbα interactions^62^ (Fig. 3d). Consistent with the BFP results, we observed a slightly higher tethered platelet density on 1261^H^-A1 (5.30 ± 0.18 × 10^−3^ μm^−2^) compared to that on 1238-A1 (4.33 ± 0.79 × 10^−3^ μm^−2^), while very few platelets tethered to 1261^L^-A1 (*n* ≤ 2; Fig. 3b and d, 1st column). Besides, the washed platelets were rolling two-fold slower on 1261^H^-A1 than 1238-A1 (a mean rolling velocity of 3.15 μm s^−1^ for 1261^H^-A1 *vs.* 6.71 μm s^−1^ for 1238-A1; Fig. 3c), whereas the rolling adhesion on 1261^L^-A1 was too weak to track. Given that the rolling velocity of individual platelet was inversely correlated with bond lifetimes measured in BFP experiments,^12,63^ these findings together demonstrated that the 1261-A1 exists in two distinct platelet binding phenotypes as opposed to the 1238-A1.

### 2.4 N-AIM sequence Q1238–E1260 makes the VWF-A1 binding phenotype robust

Notably, recombinant protein production is a sophisticated process involving multiple environmental factors with respect to a high pH buffer for protein dialysis and low temperature for storage.^65,66^ Although 1238-A1^39^ and 1261^L^*vs.* 1261^H^-A1^15^ we tested were from two different groups, they were generated in a similar protocol using *E. coli*; they may have different structure stabilities resulting in variable functional states. This also raised the hypothesis that Q1238–E1260 plays a role in stabilizing the VWF-A1 domain and protecting against environmental variations.^25^ To investigate this possibility, we challenged VWF-A1s under harsh environments, *i.e.*, low temperatures and high pH levels. 1238-A1, 1261^H^-A1, and 1261^L^-A1 were subjected to harsh environments prior to the experiments by freezing an aliquot of each construct at −80 °C or incubating with high pH (= 9.6) Tyrode buffer for 24 h before being immobilized on the microfluidic channels. In contrast to 1238-A1, which retained the same level of shear dependent platelet adhesion after being exposed to harsh environments (Fig. 3b, 2nd and 3rd columns), 1261^H^-A1 displayed a reduction in supporting platelet adhesion after harsh treatments (Fig. 3b, 2nd and 3rd columns), similar to what we previously observed with 1261^L^-A1 (Fig. 3b, 1st column). In the same microfluidic perfusion experiments, the tethered platelet density was indistinguishable for 1238-A1 under all environmental conditions (no treatment, 4.33 ± 0.79 × 10^−3^ μm^−2^; low temperature treatment, 3.39 ± 0.36 × 10^−3^ μm^−2^; high pH treatment, 3.45 ± 0.58 × 10^−3^ μm^−2^), while it reduced dramatically from 5.30 ± 0.18 × 10^−3^ μm^−2^ down to non-detectable levels for 1261^H^-A1 when low temperature or high pH level treatments were applied (Fig. 3d). In addition, the binding specificities of VWF-A1–GPIbα mediated platelet adhesion in all microfluidic perfusion experiments were well validated by a complete blockade through the addition of the anti-GPIbα antibody ALMA12 (Fig. 3b and d, 4th column). It is worth noting that the decreased binding of 1261^H^-A1 upon harsh treatments was not a result of protein aggregation, degradation, or proteolysis as evidenced by detectable adhesion frequency in BFP assays (Fig. 2e). Besides, both 1238-A1 and 1261^H^-A1 showed only one prominent protein band^39^ in SDS-PAGE analysis under reducing conditions (Fig. S2, ESI†).

**Fig. 4:**
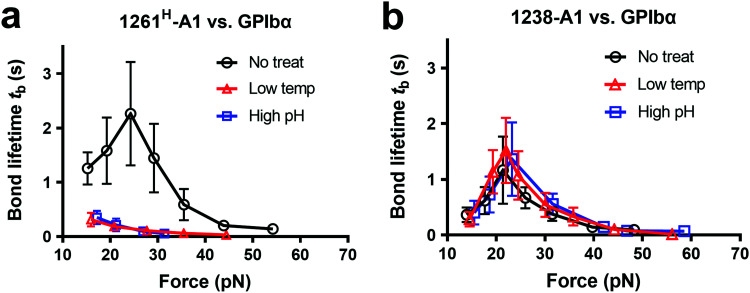
Environmental effects on GPIbα binding to 1238-A1 and 1261^H^-A1 in the absence or presence of N-AIM sequence Q1238–E1260. (a) and (b) The same environmental treatments in protein preparations were used as those in Fig. 3b. Plots of bond lifetime *vs.* force of GPIbα bonds with 1261^H^-A1 (a) and 1238-A1 (b) are generated and presented. Prior to the bond lifetime measurements, VWF-A1 beads were under the following treatments: no treatment (black circle); low temperature, frozen under −80 °C overnight and then thawed (red triangle); and high pH level (9.6) incubation with an adjusted Tyrode buffer (blue square).

To further gain biomechanical molecular insights, we measured force-dependent dissociation kinetics of single GPIbα from 1238-A1 or 1261^H^-A1 using the BFP. When treated at either a low temperature or a high pH level, 1261^H^-A1 catch bond was abolished and dissociated as a slip bond (Fig. 4a), which phenocopied 1261^L^-A1 (Fig. 2f, orange). In comparison, the 1238-A1–GPIbα bond lifetimes exhibited identical patterns (peak bond lifetime at 20–25 pN: 1.364 ± 0.660 s) under all conditions (Fig. 4b). The distinct stabilities of 1238-A1 *vs.* 1261-A1 constructs raised a possibility that Q1238–E1260, which was present in the 1238-A1 but absent in the 1261-A1 constructs, rescued the catch-bond behavior^12^ and stabilized the VWF-A1 structure regardless of the environmental factors. Notably, our results were consistent with the thermodynamic data indicating that 1261^H^-A1 is less stable than 1238-A1^15^ since treatments at a low temperature and a high pH level converted 1261^H^-A1 to 1261^L^-A1 but had no impact on 1238-A1. Together, these data demonstrated that a low temperature or a high pH level induced a transition of the VWF-A1 functional state from high (catch like) to low (slip like) binding with GPIbα.

### 2.5 C-AIM sequence P1467–N1493 interacts with N-AIM and partially blocks the VWF-A1–GPIbα binding site

**Fig. 5:**
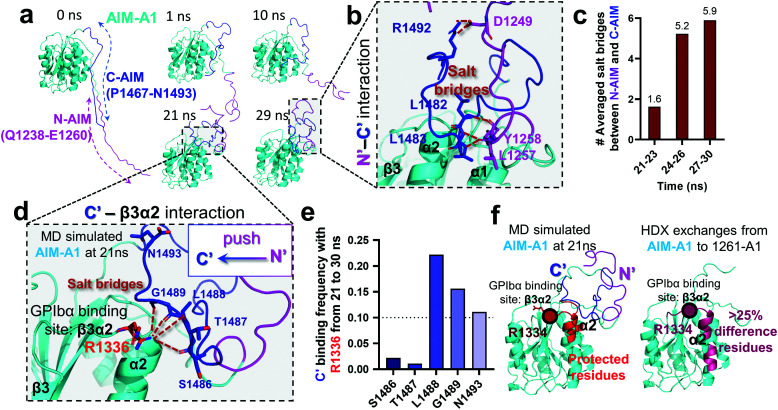
Computational modeling of the interaction of the C-AIM sequence P1467–N1493 with N-AIM and VWF-A1. (a) Sequential snapshots of free MD-simulated structures showing the AIM-A1 (cyan) interplay with N-AIM sequence Q1238–E1260 (magenta) and C-AIM sequence P1467–N1493 (blue). At *t* = 21 ns, C-AIM and N-AIM formed a joint Rotini-like structure that interacts with AIM-A1 α1/α2 helices and α1β2/β3α2 loops. (b) Snapshot of P1467–N1493 (blue) and Q1238–E1260 (magenta) interaction from the MD predicted structure at 29 ns. Note that multiple salt bridges (dark red) were formed. (c) The average number of salt bridges formed between sequence P1467–N1493 and Q1238–E1260 from 21 to 30 ns in simulation. Note that the interactions between N- and C-AIM sequences were further stabilized after 24 ns. (d) Structure of sequence P1467–N1493 interacting with β3α2 loop at 21 ns. Note that five potential salt bridges were identified. (e) The binding frequency of 5 salt bridges between P1467–N1493 and the β3α2 loop residue R1226 from 21 to 30 ns. Note that three salt bridges were likely to form in 10% frequency. (f) The protected residues (red) by sequence P1467–N1493 (blue) and Q1238–E1260 (magenta) from the MD predicted AIM-A1 structure (left red) and the HDX experiment (right pink).^41^ Residues within 7 Å to N-AIM or C-AIM in the simulation were considered protected. Note that the computational results demonstrated similar protective effects to the HDX exchanges at the α2 helix and β3α2 loop.

An HDX study suggested that the C-AIM sequence P1467–N1493 cooperates with the N-AIM in shielding VWF-A1.^13,28,41^ To further investigate this N/C-AIM cooperativity, we performed 30 ns free MD simulation for VWF-A1 with both C-AIM and N-AIM sequences (denoted as the AIM-A1; Fig. 5a, *t* = 0 ns). In the AIM-A1 simulation, the N-AIM was raised at 10 ns, rotating 60° counterclockwise, similar to the MD predicted 1238-A1 structure (Fig. 5a). It subsequently interacted with the C-AIM and formed a joint Rotini-like structure from 21 to 30 ns. Within the same period, the bonds between N- and C-AIM increased and stabilized with five salt bridges after 24 ns (Fig. 5b and c), displaying a joint Rotini-like structure formed to partially mask AIM-A1 at α1/α2 helices and α1β2/β3α2 loops. However, neither the N-AIM nor C-AIM formed stable secondary structures during the 30 ns period. In addition, we investigated the interaction between the C-AIM and the main structure of VWF-A1 (sequence D1269–D1472). From 21 to 30 ns in our free MD simulation, the C-AIM bound firmly with the β3α2 loop, which is one of the VWF-A1–GPIbα binding sites (Fig. 5d). Five salt bridges were observed between the C-AIM and the β3α2 loop at residue R1336, three of which had >10% binding frequency from 21 to 30 ns (Fig. 5e). Furthermore, we investigated the residues within 7 Å to N-AIM or C-AIM in the MD predicted AIM-A1 structure, where the VWF-A1–GPIbα binding residue R1334 was covered and protected by the C-AIM (Fig. 5f, left, dark red). Together, these findings are consistent with HDX exchange experiments between AIM-A1 and 1261-A1 (Fig. 5f, right), suggesting that N-AIM together with C-AIM have partially inhibitory effects on VWF-A1–GPIbα interaction.

## 3. Discussion

As a fascinating protein mechanosensor model, the A1 AIM flanking region was found to contribute to the force-responsive controlling of VWF activation. Our combined experimental and computational approaches demonstrated new mechano-regulation of the N-AIM sequence Q1238–E1260 and the C-AIM sequence P1467–N1493 on A1–GPIbα binding, as well as their distinct structural insights. Consistent with previous studies, 1238-A1 demonstrated unique catch bond behavior as the force first decelerated and then accelerated the dissociation of VWF-A1 bonding.^4,10,12,13^ In contrast, we found that two 1261-A1 constructs with the same sequences display distinct phenotypes: 1261^H^-A1 exhibits catch-bond behavior and higher platelet tethering in microfluidic perfusion assays, while 1261^L^-A1 exhibits slip bonds with significantly reduced platelet tethering. Intriguingly, 1238-A1 catch-bond behavior is much more stable against harsh environmental challenges. Our conclusions drawn from *in silico* analyses with respect to 1238-A1 *vs.* 1261-A1 were complemented by BFP and microfluidic perfusion experiments.

In the present study, our results are consistent with the previous findings that N-AIM,^23,27,36,67^ C-AIM,^13,28^ adjacent A2^24,26,47^ and D′D3 assembly^51^ collectively contribute to VWF autoinhibition mechanisms. Specifically, our structural analyses showed that the N-AIM Q1238–E1260 formed a Rotini-like structure covering the back face of 1238-A1 (Fig. 1e) and thereafter stabilized the α1/α6 helices (Fig. 1f). Notably, this in-*cis* A1 interaction involves the critical residues R1306 and R1450 for the sliding–rebinding mechanism (Fig. 1e), which accounts for the A1–GPIbα catch-bond behavior as previously described.^12^ Besides, C-AIM residues L1488, G1489, and N1493 can form salt-bridges with R1336 at the GPIbα binding site β3α2 loop (Fig. 5d), which most likely suppress the VWF-A1–GPIbα interaction. Last but not least, we noticed that three salt-bridges (L1257–L1483, Y1258–L1482, and D1249–R1492) were formed between C-AIM and N-AIM (Fig. 5f). This further supported the hypothesis that the two AIM sequences would form a joint Rotini-like structure, thereby autoinhibiting A1–GPIbα binding like a fastened zipper.^13^ The simulated N-AIM–C-AIM, N-AIM–α1/α6 and C-AIM–β3α2 interplay were in line with the results of HDX mass spectrometry and A108 antibody mapping^15,27^ conducted by other groups independently.

It has been long established that VWF–GPIbα catch-bond behavior well correlates with the counterintuitive high shear-dependent and shear-enhanced platelet adhesion to the VWF surface.^34,64,68,69^ Conversion of VWF–GPIbα catch bonds to slip bonds was shown to associate with von Willebrand disease mutations.^1,18,19^ Our results further demonstrated that the long N-AIM sequence in VWF-A1 (*i.e.*, 1238-A1) stabilized the catch-bond behavior, while truncating the N-AIM (*i.e.*, 1261-A1) altered the binding kinetics and resulted in the bi-variable catch-to-slip bond phenotypes. How the removal of N-AIM leads to bi-variable catch–slip bond behaviors on A1–GPIbα is an important question for future studies but we postulate multiple potential mechanisms. The first possibility is in accordance with the fact that N-AIM (residues 1238–1271) and C-AIM (residues 1459–1493) are *O*-glycosylated. Different glycosylation levels of 1261^L^-A1 and 1261^H^-A1 might account for their bi-variable bond behaviors. To this point, the seminal study by Cruz *et al.* has shown that deglycosylated VWF-A1 can mimic the physiological conditions with a slightly higher affinity than the glycosylated VWF-A1 protein, but had a similar binding capacity with platelets.^39^ Nevertheless, under different mechanical microenvironments, glycosylation may have significant contributions to the A1 folding state, thermostability,^70^ and subsequently platelet adhesive function.^39,46,65^

The second possibility may be related to two distinct α6 helix phenotypes that were found and termed as broken (1SQ0) or non-broken (1AUQ) according to the presence of ruptured α6 helical hydrogen bonds. Notably, the broken α6 helix from the original crystal structure (1SQ0) was rejoined (converted from broken to non-broken) after being linked to N-AIM in our MD simulation on 1238-A1 (Fig. 1f, left), suggesting the instability of the short N-AIM A1 structure when subjected to environmental factors (*cf.*Fig. 4). This α-helical effect finding provides a plausible structural explanation on the bi-variable binding phenotypes of 1261^L^-A1 *vs.* 1261^H^-A1, and why Q1238–E1260 could rescue the VWF-A1–GPIbα catch bond but reduce the bond lifetimes. Nevertheless, the crystal structures of 1SQ0 and 1AUQ are of resolution at 2.63 Å and 2.30 Å, respectively, which may not be enough to ascribe this helix explanation definitively.

Taken together, our study provides new mechanobiology on how N-AIM serves as a mechano-regulator of VWF activity. Given that many other mechanosensory proteins such as titin, Notch receptor, tropomyosin, *etc.*, share similar autoinhibitory features to the VWF with many of their AIMs unsolved yet,^33^ our novel approach which combines BFP, MD simulation and microfluidics represents an acceleration loop to help elucidate the regulatory mechanisms of AIMs and their associated mechanosensory structural insights.

## 4. Experimental section

### Proteins and antibodies

The recombinant monomeric 1238-A1 (residues Q1238–P1471) and 1261^L^-A1 (residues D1261–P1471) were provided by Cruz's lab, generated by *E. coli*, and purified from inclusion bodies using the same procedure as previously described.^4,39^ The proteins were then stored and shipped on dry ice under the same conditions.^4^ 1261^H^-A1 was a gift from Auton's group, generated and purified following published methods.^15,40^ All proteins were deglycosylated using *O*-glycanase.^4,15,39,40^ The binding affinity between GPIbα and glycosylated or deglycosylated VWF-A1 has been first established and validated by Cruz *et al.* in the field.^39^ They have demonstrated that the deglycosylated protein mimics the physiological conditions with a slightly higher affinity (*K*_d_ = 1.4 ± 0.4 μM) than the glycosylated protein (*K*_d_ = 4.5 ± 0.9 μM) but has a similar binding capacity with platelets. To prevent VWF-A1 from turning to its low binding state, we recommend avoiding harsh experimental environments when handling 1261-A1 or other recombinant VWF-A1 with short N-AIM sequences. In addition, the protein should be shipped on ice (instead of dry ice) to keep it near 0 °C. The protein quality of VWF-A1 constructs upon different environmental conditions was assessed by the SDS-PAGE western blot analysis followed by Coomassie Blue staining (Fig. S2, ESI†) as previously described.^39^ ALMA12 monoclonal antibody was from F. Lanza (INSERM U.311). Full-length plasma VWF (Biostate®) is manufactured from human plasma donated by New Zealand's voluntary by CSL Behring Australia. Reagents including streptavidin–maleimide (SA–MAL) were purchased from Sigma-Aldrich unless specified otherwise.

### Purification of platelets and red blood cells

All procedures involving the collection of human blood were in accordance with the Human Research Ethics Committee (HREC, project 2014/244) at the University of Sydney, and the protocol was approved by the Georgia Institute of Technology Institutional Review Board. Informed consents were obtained from human participants of this study. Specifically for this study 3 mL of venous blood was drawn from healthy donors to obtain isolated RBCs and platelets. The whole blood was initially collected in a 1 : 10 ACD buffer (6.25 g of sodium citrate, 3.1 g of citric acid anhydrous, and 3.4 g of d-glucose in 250 mL of H_2_O, pH 6.7) and centrifuged at room temperature at 900*g* for 5 min. Platelet pellets were extracted for producing washed platelets as described before.^4^

The RBCs were collected from the bottom layer of the centrifuged blood. Biotin–PEG3500–SGA (JenKem USA, TX) was covalently linked to isolated RBCs in carbonate/bicarbonate buffer (2.1 g of Na_2_CO_3_ and 2.65 g of NaHCO_3_ in 250 mL of H_2_O, pH 8.5) with 30 min incubation at room temperature.^71^ To pre-swell RBCs for the force probe used in a buffer of physiological osmolarity, the RBCs were further incubated with nystatin (Sigma-Aldrich) in N2 buffer (265.2 mM KCl, 38.8 mM NaCl, 0.94 mM KH_2_PO_4_, 4.74 mM Na_2_HPO_4_, 27 mM sucrose; pH 7.2, 588 mOsm) for 30 min at 0 °C. The modified RBCs were washed twice with N2 buffer and resuspended in N2 buffer for the BFP experiments. The prepared RBCs could be stored at 4 °C for weeks before conducting experiments.

### Functionalization of glass beads

Proteins (1238-A1, 1261^L^-A1, 1261^H^-A1, and shedded GPIbα or glycocalicin) were covalently modified with maleimide–PEG3500–NHS (MW ∼ 3500 Da; JenKem, TX) in a carbonate/bicarbonate buffer (pH 8.5). To coat maleimized proteins on glass beads, 2 μm (diameter) silanized borosilicate beads (Thermo Scientific) were first covalently coupled with mercapto-propyl-trimethoxy silane (Sigma), followed by covalently linking to both streptavidin–maleimide (Sigma) and maleimide modified proteins in monobasic/dibasic phosphate buffer (pH 6.8). The mixture would be incubated overnight and resuspended in phosphate buffer (pH 6.8) with 0.5% BSA. The specificity and functional effect were justified in the previous study.^4^ Beads were then ready for experiments. In cases of performing harsh environmental treatments, functionalized probe beads would then undergo either low temperature freezing under −80 °C overnight, then thawing, or high pH level (9.6) incubation with an adjusted Tyrode buffer for 24 h.

As previously described,^4^ we have compared three immobilization surface chemistry and constructs using glycocalicin (covalent linking by maleimide–PEG3500–NHS), full-length GPIbα (captured by the WM23 antibody), and native GPIbα (bound on the platelet membrane). The data showed indistinguishable catch-bond behaviors in force *vs.* bond lifetime curves, indicating that the VWF-A1–GPIbα dissociation kinetics is independent of these immobilization chemistries.^4,10,47^

### BFP experiments

The BFP setup^58^ and experimental procedures to study the A1–GPIbα interaction has been described in detail.^4,10,44,45,64^ Briefly, isolated RBCs were pretreated with Biotin–PEG3500–NHS (MW ∼ 3500 Da; JenKem, TX), which enables covalent binding with the SA coupled probe beads to form an ultrasensitive spring (Fig. 2a, top). The stiffness of the RBC (*k*_RBC_) can be determined by the radii of the orifice (*R*_p_), the probe bead (*R*_c_) and RBC (*R*_0_) when the aspirated tongue length (*L*_p_) of the RBC is equal to *R*_p_:^34,72^1
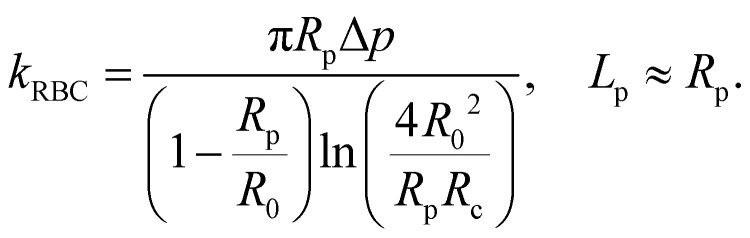
The pressure applied to the RBC is precisely controlled by our homemade manual water manometer so that the spring constant of the RBC can be modified to 0.3 pN nm^−1^ by adjusting the height difference between the water level in the reservoir and the tip of the micropipette.^34,73^ As a result, the force applied to the probe bead can be interpreted *via* the deflection of the RBC. During each experiment cycle (Fig. 2b), the target bead was driven by a piezo actuator and approached the probe bead (VWF-A1 coated bead) with a 20 pN compressive force for a certain contact time (0.2 s by default) to allow for bond formation. The target bead was then retracted for adhesion detection in the force regime of 10–70 pN (Fig. 2b). During the retraction phase, a ‘bond’ event was signified by tensile force (Fig. 2d), but no tensile force was detected in a ‘no bond’ event (Fig. 2c). For the adhesion frequency assay, ‘bond’ and ‘no bond’ events were enumerated to calculate adhesion frequency (*P*_a_) in 200 repeated cycles for each probe–target pair. In the force-clamp assay, the target bead was held at the desired force, termed clamp force (Fig. 2b and c, red dashed line), to wait for bond dissociation and return to the original position to complete the cycle. Bond lifetimes were measured from the instant when the force reached the clamp force level to the instant when the bond dissociated. To ensure single-bond measurement, VWF-A1 site densities on the probe beads were controlled so that a low adhesion frequency was obtained. It is worth mentioning that according to the Poisson Statistics,^74^ in rare (<20%) bond events, the probe bead was pulled by a single VWF-A1–GPIbα bond in most (>89%) cases.^10^

### Microfluidic channel perfusion assays

Washed platelets were perfused in a PDMS channel (channel dimension: 200 μm in width × 70 μm in height) at a *γ* = 800 s^−1^ wall shear rate, then the platelet transient adhesion assays were performed as previously described.^4,10,12^ VWF-A1s or full-length plasma VWF (50 μg mL^−1^) were directly coated onto the bottom coverslip by physical absorption. Tethered platelets at *γ* = 800 s^−1^ are monitored with confocal microscopy with differential interface contrast imaging (a Nikon A1R confocal microscope with a ×60 water objective and a ×1.5 Leica objective lens). A 54 μm × 54 μm selected ROI was applied offline in ImageJ (1.53c; Wayne Rasband, National Institutes of Health) for counting platelet tethering over the 30 s perfusion. The tethered platelet density was calculated as the number divided by ROI area in the focal plane. As previously described, platelet mean rolling velocities were measured as cell displacement divided by the tracking interval.^12^ Only platelets that traveled more than one cell distance (2 μm) within 1 frame (1 s) were analyzed. For experiments under harsh environmental treatment, VWF-A1 aliquots were pretreated as previously described and immobilized on the surface of a microfluidic channel. In certain experiments, the anti-GPIbα monoclonal antibody (clone ALMA12; 10 μg mL^−1^) was added to block GPIbα mediated platelet adhesion.

### Molecular dynamics simulations

We performed MD simulations on 1238-A1 (residues Q1238–P1466) and AIM-A1 (residues Q1238–N1493). To get the N-AIM and C-AIM structures, the entire VWF sequence was obtained from the NCBI protein database (UniProtKB/Swiss-Prot: P04275.4; Fig. 5a). PyMOL (version 2.5.1 by Schrödinger) was used to create amino acid connections. The linked N-AIM and C-AIM were extended from the N-terminal and C-terminal of the existing VWF-A1 structure (PDB: 1SQ0). Manual adjustments were optional to avoid atom collisions. Hydrogens were removed before MD simulation.

For simulations of 1238-A1 and AIM-A1, each sequence was neutralized with Na^+^ or Cl^−^ ions then soaked into a 1.0 nm larger water box under periodic boundary conditions. The rebuilt structures served as the starting point for MD simulations using GROMACS with the CHARMM27 force field and TIP3P water model.^75,76^ The system was first energy-minimized to maximum force <1000 kJ mol^−1^ nm^−1^, then subjected to equilibration for 0.1 ns under *NVT* followed by 0.1 ns under an *NPT* ensemble with 300 K temperature and ambient pressure. The equilibrated system at 0.2 ns was taken for further simulations. Each system was recorded every 100 ps with a 2 fs time step. Protected residues in the predicted structures were defined as residues within 7 Å to any N-AIM or C-AIM atoms. The distance measurements were performed in Pymol.

## Author contributions

L. A. J. and Y. C. Z. wrote and revised the paper, performed research and analyzed data, conducted analysis and interpretation of data, and designed research; H. W. and Y. W. wrote and revised the paper, and performed analysis and interpretation of data; J. L. contributed to the preliminary data and analysis. L. A. J. and Y. C. Z. are equal first authors.

## Conflicts of interest

The authors declare no conflict of interest.

## Supplementary Material

CB-003-D2CB00010E-s001

CB-003-D2CB00010E-s002

CB-003-D2CB00010E-s003
