# Supplementary figures and images for: The N-terminal autoinhibitory module of the A1 domain in von Willebrand factor stabilizes the mechanosensor catch bond
Source: RSC Chem Biol. 2022 Apr 7;3(6):707–20. doi: 10.1039/d2cb00010e (PMC9175105; doi:10.1039/d2cb00010e)

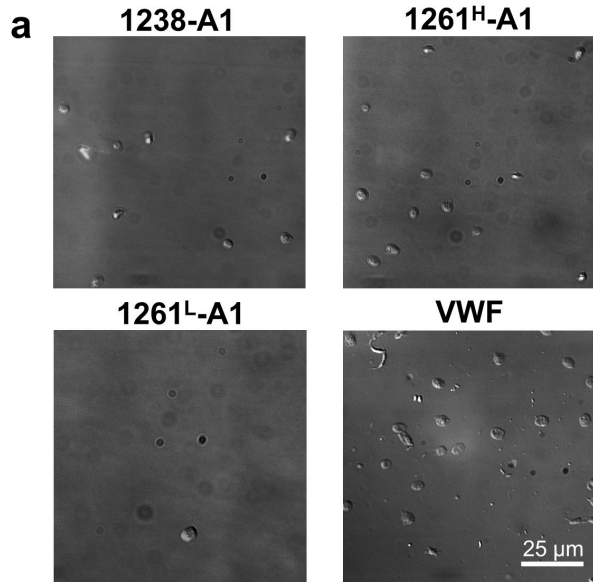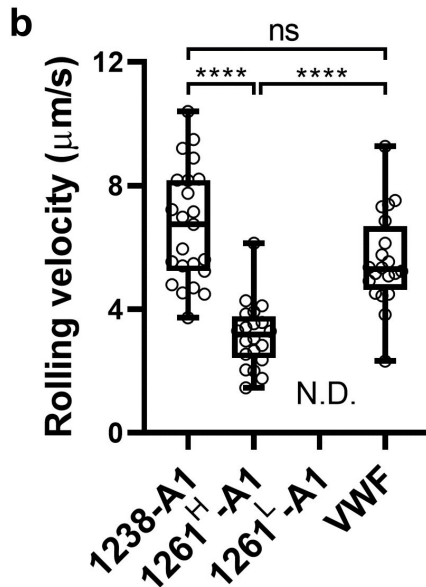

Supplement: CB-003-D2CB00010E-s001 [file CB-003-D2CB00010E-s001.pdf]

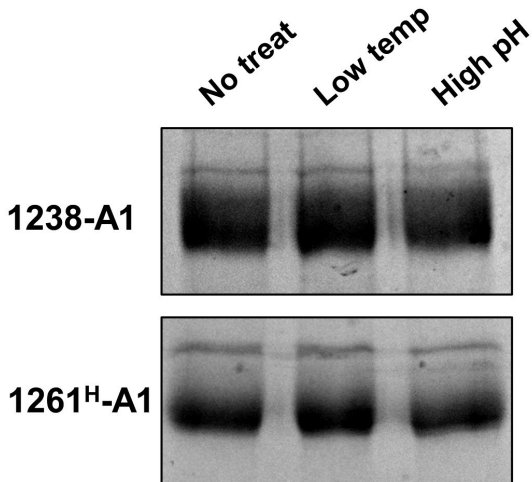

Supplement: CB-003-D2CB00010E-s002 [file CB-003-D2CB00010E-s002.pdf]
