# Supplementary material for: The N-terminal autoinhibitory module of the A1 domain in von Willebrand factor stabilizes the mechanosensor catch bond
Source: RSC Chem Biol. 2022 Apr 7;3(6):707–20. doi: 10.1039/d2cb00010e (PMC9175105; doi:10.1039/d2cb00010e)
Supplement: CB-003-D2CB00010E-s003 [file CB-003-D2CB00010E-s003.pdf]

Electronic Supplementary Material (ESI) for RSC Chemical Biology.
This journal is © The Royal Society of Chemistry 2022

Supplementary Information

Title: N-terminal Autoinhibitory Module of A1 Domain in von Willebrand Factor Stabilizes the Mechanosensor Catch Bond

Authors and Affiliations:

Yunduo Charles Zhao[1,2†], Haoqing Wang[1,3], Yao Wang[1,4], Jizhong Lou[5,6,7], Lining Arnold Ju[1,2,3,8†*],

[1]*School of Biomedical Engineering, Faculty of Engineering, The University of Sydney, NSW, Australia.*
[2]*Charles Perkins Centre, The University of Sydney, NSW Australia*
[3]*Heart Research Institute, NSW, Australia*
[4]*Cellular and Genetic Medicine Unit, School of Medical Sciences, University of New South Wales, NSW, Australia*
[5]*Key Laboratory of RNA Biology, CAS Center for Excellence in Biomacromolecules, Institute of Biophysics, Chinese Academy of Sciences, Beijing, China*
[6]*University of Chinese Academy of Sciences, Beijing, China*
[7]*Bioland Laboratory (Guangzhou Regenerative Medicine and Health Guangdong Laboratory), Guangzhou, Guangdong, China*
[8]*Coulter Department of Biomedical Engineering, Georgia Institute of Technology, Atlanta, GA, United States*
[†]*These authors contribute equally.*
[*]*Corresponding author:*
Lining Arnold Ju. Email: Arnold.ju@sydney.edu.au

Figure S1~S2

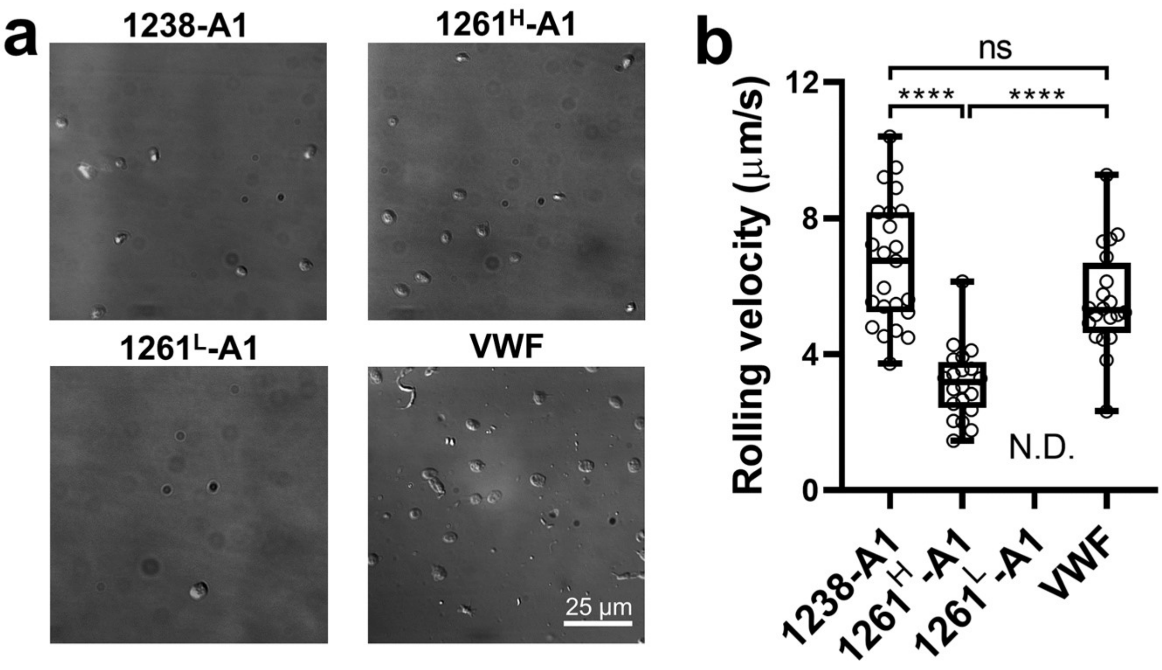

Figure S1. Platelet tethering on the surface of flow chamber coated with VWF-A1 variants or full-length VWF. a) Representative snapshots of tethered platelets (bright white objects) in a microfluidic channel, at fixed wall shear rate γ = 800 s^{-1}, pre-coated with 1238-A1, 1261^{H}-A1, 1261^{L}-A1 or full-length plasma VWF. The photomicrographs depict the platelets tethered and were rolling on the surface during perfusion and each represents experiments with two different platelet donors. b) Platelets rolling velocity ($n \geq 20$) during perfusion. Data were presented as box plots. N.D. = not detectable. **** = $p < 0.0001$; ns = not significant, assessed by unpaired, two-tailed Student's *t*-test.

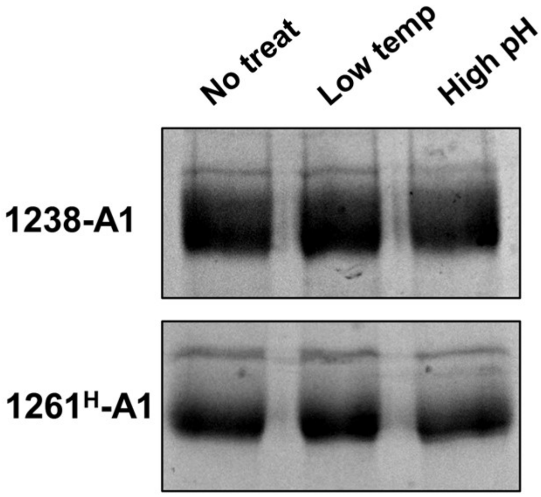

Figure S2. 1238-A1 and 1261^{H}-A1 with indicated environmental factors were analyzed under reducing condition by SDS-PAGE (12.5%) and Coomassie Blue staining.
